# Efficacy of *Lactobacillus* spp. Interventions to Modulate Mood Symptoms: A Scoping Review of Clinical Trials

**DOI:** 10.3390/ijms26168099

**Published:** 2025-08-21

**Authors:** Diego Fernández-Rodríguez, María Consuelo Bravo, Marcela Pizarro, Pablo Vergara-Barra, María José Hormazábal, Marcell Leonario-Rodriguez

**Affiliations:** 1 Escuela de Nutrición y Dietética, Facultad de Medicina y Ciencias de la Salud, Universidad Mayor, Santiago 8580745, Chile; diego.fernandezr@mayor.cl (D.F.-R.); marcela.pizarro@mayor.cl (M.P.); 2 Academia Científica de Estudiantes de Nutrición y Dietética, Facultad de Medicina y Ciencias de la Salud, Universidad Mayor, Santiago 8580745, Chile; 3 Departamento de Nutrición y Dietética, Facultad de Farmacia, Universidad de Concepción, Concepción 4070386, Chile; mbravo2016@udec.cl; 4 Facultad de Psicología, Universidad del Desarrollo, Concepción 4070386, Chile; pablovergara@udd.cl; 5 Programa de Doctorado en Ciencias, Mención Biología Celular y Molecular Aplicada, Universidad de La Frontera, Temuco 4811230, Chile

**Keywords:** gut–brain axis, gut microbiota, mood disorders, probiotics, *Lactobacillus* spp.

## Abstract

Probiotics containing *Lactobacillus* spp. have demonstrated immunological and gastrointestinal benefits and may aid in recovery from mood disorders. However, evidence of their mood-modulating efficacy remains inconsistent. Aim: To analyze the efficacy of probiotic interventions with *Lactobacillus* spp. in modulating mood in humans. A scoping review was conducted following the PRISMA guidelines. A systematic search of the PubMed and Scopus databases was performed using nine Boolean combinations of the terms “mental”, “mental diseases”, “mental disorders”, “gastrointestinal microbiome”, “gut microbiome”, “gut microbiota”, and “lactobacillus”. The search was limited to clinical trials published in English and limited to ten years of publication. Eligible studies met the following criteria: (a) probiotic interventions in adults, with or without mood disturbances; (b) the use of *Lactobacillus* spp., either alone or in combination; (c) mood assessment instruments applied pre- and post-intervention; and (d) reporting of probiotic concentrations. Trials involving populations with other psychiatric or neurological diagnoses or those combining probiotics with additional mood-modulating nutrients were excluded. From 3291 records, 17 clinical trials met the inclusion criteria. Data extracted included the author, year, population, country of origin, probiotic strain(s), dosage, intervention mode and duration, and outcomes related to the microbial composition, biomarkers, and microbial metabolites. Trials were categorized by probiotic type (single vs. multi-species) and participant profile (healthy individuals and those with depressive symptoms or specific physiological conditions). Preliminary evidence from single-strain interventions, particularly high-dose *L. plantarum* administered for ≥8 weeks, suggests potential improvements in anxiety, sleep quality, and inflammatory biomarkers. Multi-species formulations yielded reductions in depressive symptoms and changes in neurobiological markers. Nonetheless, substantial heterogeneity in strains, dosages, durations, and outcome measures limited cross-study comparisons. *Lactobacillus* spp. interventions show promising mood-modulating potential, especially with specific strains and prolonged administration. Standardized protocols, rigorous controls, and clearly defined clinical cohorts are needed to establish robust, evidence-based recommendations.

## 1. Introduction

The gut–brain axis is a bidirectional communication network linking the central nervous system, the enteric nervous system, and the gut microbiota (GM). Over the past two decades, the microbial community inhabiting the gastrointestinal tract has been recognized as playing a fundamental role in the homeostasis of this axis [1]. This modulation is mediated by immunological, endocrine, and neuronal pathways, including regulating mood and stress responses in humans. This relationship is founded on the influence of the GM on neurotransmitter metabolism and its concrete role in systemic inflammation, a key factor in the pathophysiology of psychiatric disorders [2].

Several observational studies have demonstrated that alterations in the composition of the GM, referred to as dysbiosis, are associated with a variety of mental health conditions, including depression, anxiety, and chronic stress [3,4,5]. This dysbiosis is characterized by a reduction in beneficial bacteria such as Lactobacillus and Bifidobacterium, accompanied by an increase in potentially pathogenic genera, including Clostridium, Desulfovibrio, and particular species of Proteobacteria [6].

Notably, a decrease has been observed in short-chain fatty acid (SCFA)-producing species such as *Faecalibacterium prausnitzii* and *Roseburia* spp., which may compromise intestinal barrier integrity and promote low-grade systemic inflammation. In addition, an increase in Eggerthella, Parabacteroides, and Alistipes has been associated with dysbiotic profiles in individuals with depression. These alterations in bacterial composition could affect neurotransmitter production and metabolism and regulate the gut–brain axis, thereby contributing to the pathophysiology of mood disorders [7,8]. In this context, probiotics have emerged as a potential therapeutic strategy to address mood disturbances. The evidence is robust, indicating that probiotics are effective in modulating the intestinal microbiota composition and improving mood [9,10].

One of the most extensively studied genera in human health is Lactobacillus, a group of bacteria renowned for their probiotic properties—particularly their ability to produce lactic acid, which acidifies the intestinal environment and promotes the growth of beneficial microorganisms [11]. This metabolic activity, combined with the production of neurotransmitters and other bioactive compounds, can directly influence gut–brain communication. It has been demonstrated that particular species of this genus can synthesize neurotransmitter precursors such as serotonin and dopamine, which play pivotal roles in regulating emotional states and stress responses. Moreover, some studies suggest anti-inflammatory effects, which may help to mitigate the associated neuroinflammation. The underlying mechanisms of these effects include the modulation of the immune system, reductions in intestinal permeability, and involvement in autonomic nervous system regulation [12,13].

However, the variability in outcomes suggests that not all *Lactobacillus* spp. and strains exert the same effects, underscoring the need for more targeted and systematic investigations. Employing probiotics of this genus as a therapeutic intervention in mental health raises several questions that must be addressed. Among these is determining optimal dosages and the conditions under which *Lactobacillus* spp. can elicit beneficial effects. Some clinical trials have reported promising results, indicating that the administration of *Lactobacillus* spp. can reduce cortisol levels—a biomarker of stress—and alleviate anxiety symptoms in patients with mood disorders. However, these interventions are often accompanied by probiotic formulations supplemented with vitamins or other critical nutrients, potentially confounding the specific contribution of the *Lactobacillus* genus [14,15]. The species employed, the administered dosage, the treatment duration, and the individual psychosocial characteristics of the participants constitute critical factors that preclude the generalization of *Lactobacillus* spp.’s effects in this field. Accordingly, the objective of the present study was to evaluate the effectiveness of interventions with *Lactobacillus* spp. in modulating mood in human subjects.

## 2. Methods

The present scoping review was conducted according to the Preferred Reporting Items for Systematic Reviews and Meta-Analyses (PRISMA) guidelines for scoping reviews [16]. The research question addressed was “Are probiotic interventions with *Lactobacillus* spp. effective in modulating mood symptomatology in human subjects?” The literature search was conducted in the PubMed and Scopus databases using the terms “mental”, “mental diseases”, “mental disorders”, “gastrointestinal microbiome”, “gut microbiome”, “gut microbiota”, and “lactobacillus”, combined with the Boolean operator “AND” to generate nine distinct search queries, each comprising three concatenated terms. The search was restricted to clinical trials published to date in English and limited to ten years of publication. It was performed independently by two investigators (D.F. and M.P.), with a third author (M.L.R.) resolving any discrepancies in article selection.

The inclusion criteria were (a) probiotic intervention studies in adult humans with or without mood disturbances; (b) interventions employing *Lactobacillus* spp., either alone or in combination with other genera; (c) studies that assessed mood symptomatology using validated instruments both before and after the intervention; and (d) studies reporting the concentrations of the probiotics administered. Conversely, we excluded studies involving populations with other mental or neurological diagnoses and any trials in which the probiotic regimen included additional nutritional components known to modulate mood.

**Figure 1:**
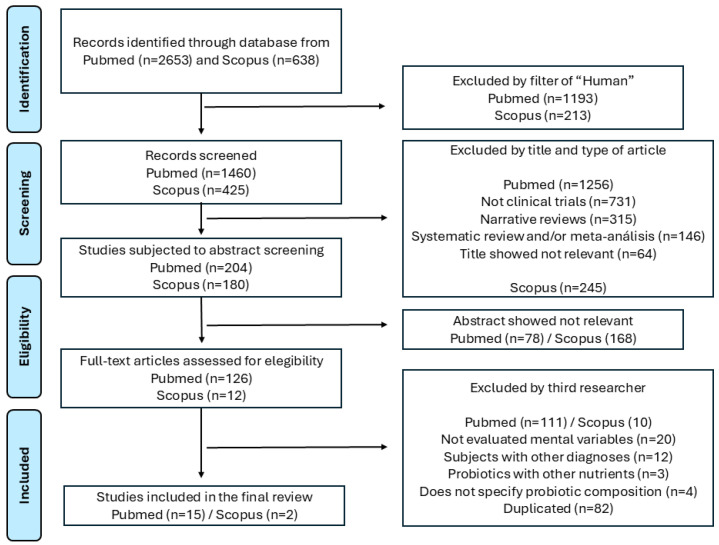
PRISMA flow diagram process for the scoping review.

All relevant data were extracted according to the study objectives, including the first author’s name, year of publication, study population and origin, probiotic strain(s), concentration, intervention modality and duration, and outcomes at the levels of the microbiota composition, biomarkers, and microbial metabolites. An initial yield of 2653 records from PubMed and 638 from Scopus was retrieved. After applying the “Humans” filter, 1460 PubMed and 425 Scopus records remained for title and article type screening. After excluding non-clinical trials, narrative reviews, systematic reviews, and/or meta-analyses, and studies with irrelevant titles, 204 PubMed and 180 Scopus records proceeded to abstract screening. Of these, 78 PubMed and 168 Scopus abstracts were deemed irrelevant, yielding 126 PubMed and 12 Scopus full-text articles for eligibility assessment. The application of predefined exclusion criteria by a third researcher, including the removal of duplicates (n = 82), studies without specified probiotic compositions (n = 4), those on the use of probiotics in combination with other nutrients (n = 3), those with subjects with alternative diagnoses (n = 12), and studies not evaluating mental health outcomes (n = 20), resulted in the exclusion of 111 PubMed and 10 Scopus articles. Ultimately, 15 PubMed and 2 Scopus studies met all eligibility criteria and were included in the final review (Figure 1). These studies were collated into an Excel^®^ matrix for critical appraisal in line with the review’s objectives.

Following study selection and critical data extraction, we assessed the risk of bias in each included trial under the Cochrane Handbook’s recommendations. Specifically, we applied the Cochrane Risk of Bias 2.0 tool across six domains: random sequence generation, allocation concealment, blinding of participants, blinding of outcome assessors, incomplete outcome data, and selective reporting. Two reviewers independently rated each domain as “low”, “some concerns”, or “high” risk; any discrepancies were resolved through discussion until a consensus was reached.

## 3. Results

### 3.1. Characteristics of the Included Studies

**Figure 2:**
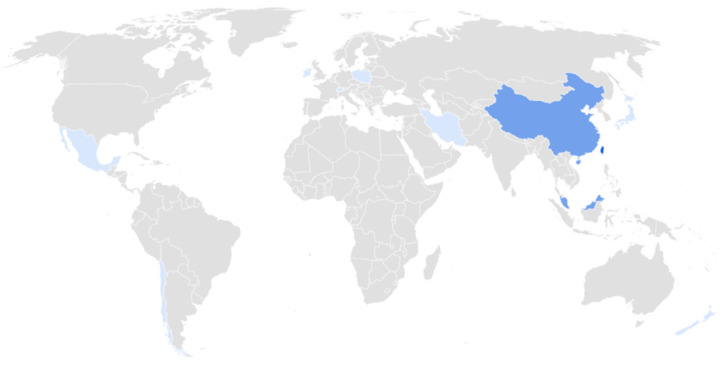
Geographic distribution of clinical trials analyzed. The map uses shades of blue to represent the concentration of studies by origin, indicating a majority proportion from the Asian continent, particularly from China.

Of the 17 selected studies, the majority were conducted in Asian populations (58.8%), followed by work in European cohorts (23.5%), with minimal representation from American and Oceanian participants (11.8 and 5.9%, respectively). Still, African populations have reported no trials (Figure 2). Regarding the intervention characteristics, 70.6% of the studies employed probiotic formulations exclusively containing *Lactobacillus* spp., whereas the remainder used multi-species preparations. The dosages varied markedly, ranging from 1 × 10^8^ to 3 × 10^10^ CFU. Overall, the studies consistently administered the probiotic as a standalone treatment or an adjunct to standard care. Delivery vehicles included capsules, powdered sachets, and liquid beverages, which were administered daily for 4, 8, or 12 weeks; notably, two interventions were extended to 6 months. Participants spanned healthy volunteers, individuals with specific physiological or pathophysiological conditions, and patients diagnosed with major depressive disorder.

### 3.2. Efficacy of Formulations Exclusively Containing Lactobacillus spp.

When the effects of probiotic interventions consisting exclusively of *Lactobacillus* spp. were examined in healthy populations (Table 1), only three studies were identified, conducted in Irish, Japanese, and Mexican cohorts, published in 2017, 2019, and 2021, respectively. In the four-week trial employing *Lactobacillus rhamnosus*, no significant differences were observed versus a placebo in mood measures, the GM composition, or related biomarkers [17]. Conversely, in an intervention spanning 24 weeks with nearly double the concentration of *Lactobacillus gasseri*, significant reductions in anxiety levels were observed versus a placebo. These effects were accompanied by improvements in the GM composition, particularly in the abundance of *Bifidobacterium* and *Streptococcus* spp. Additionally, participants receiving the probiotic exhibited significantly improved sleep quality, gastrointestinal symptomatology, perceived stress, and irritability compared with the control group [18]. A more recent study involving university students from Mexico evaluated a probiotic beverage containing *Lactobacillus plantarum*, *Lactobacillus paracasei*, and *Lactobacillus brevis* over 8 weeks. The intervention significantly decreased cognitive–emotional distress, as measured by the Systemic Cognitivist Inventory (SISCO). It was associated with a notable increase in Bacteroidetes and Firmicutes abundance [19].

Six studies were identified concerning outcomes in populations with specific physiological and pathophysiological conditions (Table 2); only one was conducted in New Zealand, while the remainder involved Asian cohorts. These interventions employed, individually and in combination, *Lactobacillus acidophilus*, *Lactobacillus paracasei*, *Lactobacillus plantarum*, and *Lactobacillus rhamnosus*. In the trial assessing *Lactobacillus rhamnosus* in 380 pregnant women in New Zealand, the administration of 6 × 10^10^ CFU resulted in significant improvements in postpartum depression and anxiety metrics at six months after delivery [20]. When evaluating the efficacy of *Lactobacillus plantarum*, three studies employed probiotic formulations containing only this species, in cohorts of individuals suffering from stress [21], chronic insomnia [22], and anxious students [23]. In these studies, interventions lasting one month or less resulted in significant improvements in mood compared to the placebo. Enhancements in sleep quality were also reported; however, distinct metrics were employed across trials. Among patients with insomnia, reductions were observed in fatigue levels, brainwave activity, and awakenings during the deep sleep stage. The initiative conducted on 103 stressed adults from Malaysia demonstrated significant improvements across multiple domains of the DASS-42 scale, including depression, anxiety, and stress. Moreover, the IFN-γ and TNF-α levels significantly decreased and were correlated with reductions in DASS42 scores. Regarding the gut microbiota composition, only Zhu et al. reported the restoration of bacterial genera that are critical for intestinal homeostasis, including Bacteroides, Bifidobacterium, Prevotella, and Roseburia.

Regarding the other two studies, both conducted in populations with gastrointestinal disturbances, the first involved constipated patients who consumed *Lactobacillus paracasei* (LcS) [24], and the second involved patients with irritable bowel syndrome who received the species in combination with *Lactobacillus acidophilus* [25]. In both cases, no significant mood improvements versus the placebo were observed; however, LcS consumption led to increases in Adlercreutzia, Megasphaera, and Veillonella and decreases in Rikenellaceae, Sutterella, and Oscillibacter. Furthermore, the IL-6 levels were significantly reduced. In the IBS population, probiotic intake increased serotonin levels, suggesting a modulatory effect on neurotransmitter pathways despite the absence of changes in mood outcomes.

Regarding the efficacy of Lactobacillus spp. in individuals with major depressive disorder (Table 3), three studies were identified, all of which administered *Lactobacillus plantarum* as a monotherapy. Each intervention lasted eight weeks, with capsules dosed twice daily; however, the Polish study employed a concentration of 1 × 10^9^ CFU [26], whereas the two Taiwanese trials used 3 × 10^10^ CFU [27]. Only the study by Chen et al. reported significant reductions in depressive symptomatology across two distinct rating scales, which were markedly associated with shifts in bacterial taxa linked to improved mood metrics and with its counterpart. Moreover, a correlation was observed between the detected Lactobacillus levels and markers of depressive symptomatology. Conversely, the study by Rudski et al. did not report mood-related effects; however, it demonstrated significant alterations in tryptophan metabolism biomarkers and improvements in cognitive test performance. In the most recently published study, no outcomes were reported in any domain [28].

### 3.3. Efficacy of Multi-Species Formulations Containing Lactobacillus spp.

Only two studies were identified when assessing the effects of combined species interventions in healthy populations (Table 4), exhibiting substantial methodological heterogeneity. The first, published in 2022, was conducted in a small Swedish cohort [29], whereas the second was performed in 135 Chilean subjects [30]. Concerning the interventions, both studies employed the same concentration and duration of treatment; however, the South American trial utilized *Lactobacillus helveticus* and *Bifidobacterium longum*, whereas the European study included *Lactobacillus plantarum* in addition to the species mentioned earlier. Neither publication demonstrated significant effects on mood symptomatology or associated biomarkers, and these trials did not assess the intestinal microbiota composition.

Regarding individuals with depressive symptomatology (Table 5), three studies were identified, each employing different probiotic species yet all demonstrating tangible effects on mood. The first study, conducted in an Iranian cohort and using a two-species intervention (*Lactobacillus helveticus* and *Bifidobacterium longum*), reported significant reductions in Beck Depression Inventory scores, together with the modulation of biomarkers associated with tryptophan metabolism [31]. The second study, conducted in a Korean cohort receiving *Lactobacillus reuteri* and *Bifidobacterium adolescentis*, reported significant improvements on the Beck Depression Inventory-II and reduced interleukin-6 levels. Improvements in depressive symptoms correlated with family-level abundances of genera such as Lactobacillus and Bifidobacterium, and enhancements in sleep quality were also observed [32]. Finally, in the study evaluating the efficacy of a probiotic formulation containing seven bacterial species from three different genera, significant improvements versus a placebo were reported on the Hamilton Depression Rating Scale, accompanied by the preservation of bacterial diversity metrics. These outcomes were associated with an increased abundance of the genus Lactobacillus [33].

**Figure 3:**
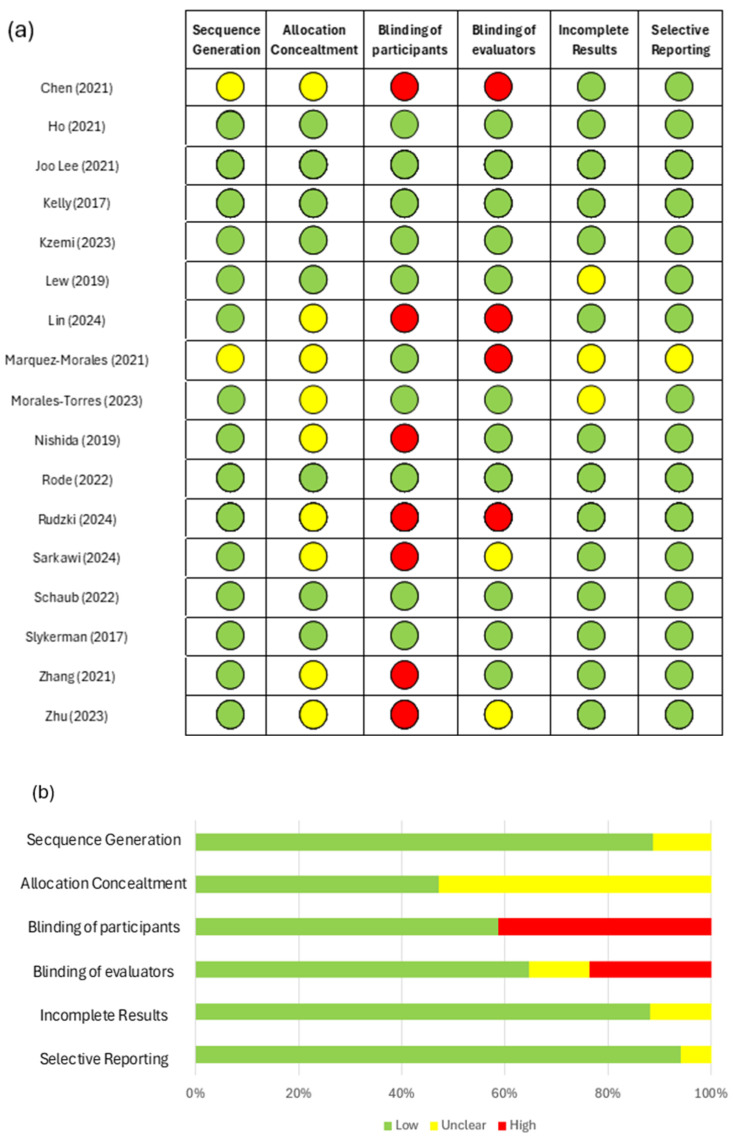
Risk of bias assessment in clinical trials using *Lactobacillus* spp. for mood modulation. Panel (**a**) presents the individual risk of bias assessment for 17 included studies across six key methodological domains [17,18,19,20,21,22,23,24,25,26,27,28,29,30,31,32,33]. Panel (**b**) summarizes the proportion of studies rated as low (green), unclear (yellow), or high risk (red) in each domain. The most frequently observed limitations were related to the blinding of participants and outcome assessors.

The risk-of-bias appraisal (Figure 3) demonstrated consistently favorable ratings for some domains and greater uncertainty or concern in others. In panel (a), nearly all 17 trials were judged as “low risk” for sequence generation, with only one study rated as “unclear”. Allocation concealment was also predominantly of low risk, although three studies raised “some concerns”, and one was deemed of high risk. In contrast, the blinding of participants showed more variability: six trials were rated as having a high risk, five unclear, and the remainder of low risk. The blinding of outcome assessors likewise exhibited substantial high risk judgments (seven studies) and a few unclear ratings, with the rest at a low risk. Importantly, all studies were rated as having a low risk for incomplete outcome data, indicating that attrition was adequately addressed. Finally, selective reporting was of low risk in most trials, with only two unclear studies. Panel (b) summarizes these findings quantitatively, illustrating that the sequence generation and incomplete data domains achieved over 90% low risk ratings, whereas the blinding domains showed approximately 40–45% high risk ratings and 5–10% unclear judgments, and selective reporting had around 12% uncertainty.

## 4. Discussion

This review identifies key trends and notable gaps in the current literature on using *Lactobacillus* spp. for mood modulation. Chief among these is the absence of studies conducted outside of Asian and European populations, representing a significant shortfall that limits the generalizability of findings across diverse cultural, genetic, and environmental contexts. This pattern reflects a broader trend in clinical research on the human gut microbiome, which remains heavily dominated by high-income countries [34]. This disparity reflects the limited availability of research funding in low- and middle-income regions and the absence of the technological infrastructure required for advanced sequencing studies [35], underscoring the need to promote a more inclusive and representative scientific agenda.

There is a pronounced interest in elucidating the isolated effects of species within the genus under study for the types of interventions analyzed. However, the growing number of publications investigating multi-genus combinations reflects the expanding exploration of potential synergistic interactions among multiple species. Discussion of these variables has emerged as a prominent topic in the literature. For example, Timmerman et al. note that a well-designed combination of species yields superior outcomes in various animal models, while emphasizing that not all mixtures will necessarily produce synergistic effects [36]. Along these lines, it has also been demonstrated that variations in the probiotic manufacturing process can yield divergent impacts, even when the same strains are employed [37]. Moreover, a systematic review concluded that the number of strains does not guarantee efficacy, and strain selection should be grounded in published clinical evidence since multi-species formulations do not necessarily confer greater benefits than single-strain products [38]. This latter premise aligns with our findings, which demonstrate efficacy in single- and multi-species formulations, suggesting that effectiveness is mediated by the specific clinical context rather than by the number of species.

From this premise, it can be hypothesized that efficacy is mediated by the pathophysiological processes experienced by individuals with mood disturbances. Evidence indicates that exposure to stressors induces the central expression of tumor necrosis factor-alpha, interleukin-6, and interleukin-10 [39,40], as observed in two studies evaluated in this review [22,32]. Furthermore, various amines secreted in the intestine promote increased intestinal permeability and the development of systemic and local inflammation. This hyperpermeable state facilitates the translocation of bacterial components, such as lipopolysaccharides, which induce the expression of proinflammatory cytokines within the central nervous system. Together, these three processes create a vicious cycle that, in the absence of treatment, suppresses the expression of brain-derived neurotrophic factors, thereby contributing to the pathophysiology of major depressive disorder [41,42]. Specifically, patients develop a state of intestinal dysbiosis accompanied by neuropathological alterations such as synaptic defects, demyelination, aberrant neurogenesis, and disrupted neurotransmitter release. In this context, the modulation of dysbiosis with probiotic formulations mitigates bacterial translocation and attenuates both peripheral and central inflammation. Consequently, this intervention reduces the magnitude of the pathophysiological processes, thereby improving the clinical symptomatology. Indeed, evidence demonstrates that *Lactobacillus* spp. increase the abundance of specific taxa that regulate barrier permeability and elevate the concentrations of SCFAs [43]. These bacterial metabolites have been identified as biological agents capable of modulating the proinflammatory cytokine levels and brain-derived neurotrophic factor concentrations [44,45].

Another mechanism identified in the studies analyzed involves the modulation of tryptophan metabolism via the kynurenine pathway. Under conditions of psychological stress or inflammation, upregulated activity of the enzyme indoleamine 2,3-dioxygenase or tryptophan 2,3-dioxygenase shifts the conversion of tryptophan toward kynurenine at the expense of serotonin synthesis, a process closely linked to depressive symptomatology. Once formed, peripheral L-kynurenine crosses the blood–brain barrier and is metabolized into either kynurenic acid or quinolinic acid, depending on the prevailing enzymatic activity. The activation of these enzymes thus exerts a dual effect: it reduces tryptophan’s availability for serotonin production and increases the generation of neurotoxic or neuroprotective metabolites. Reversing this shift diminishes kynurenine pathway flux, increasing tryptophan’s bioavailability for serotonin synthesis and thereby enhancing the modulatory role of this amine and its downstream metabolites in mood regulation, as evidenced by three of the studies included in this review [25,26,31].

Moreover, and of particular interest, two studies demonstrated that the administration of *Lactobacillus* spp. improved sleep quality by enhancing cerebral activity and parameters associated with rapid eye movement sleep [14,32]. This is consistent with published evidence indicating that, based on isolated studies and even a meta-analysis, *Lactobacillus* spp. could plausibly improve sleep quality [46,47]. Regarding the underlying mechanism, studies suggest that rapid eye movement sleep is essential in regulating emotional reactivity, consolidating emotional information, and reconsolidating memories, particularly those of a negative nature [48]. In this context, models have proposed that, during healthy rapid eye movement sleep, the locus coeruleus is silenced, reducing noradrenergic activity and promoting synaptic plasticity within limbic circuits [49]. Indeed, these circuits are directly involved in the regulation of mood, the pathophysiology of depression, and the emergence of suicidal tendencies [50], which has been corroborated by a recently published systematic review of human studies [51].

**Figure 4:**
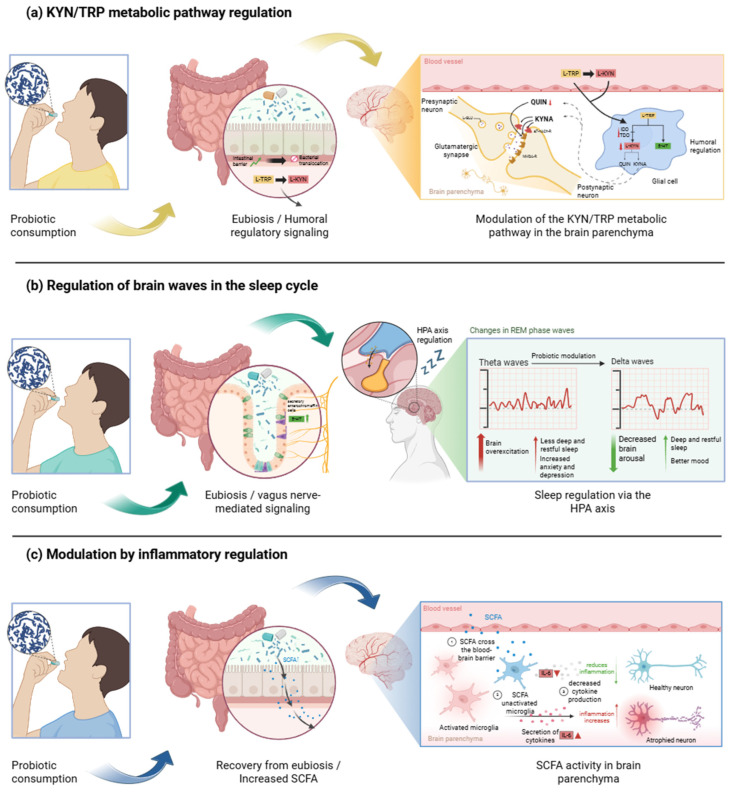
Proposed mechanisms underlying the psychobiotic effects of *Lactobacillus* spp. interventions. (**a**) Modulation of tryptophan metabolism through the kynurenine and serotonin (5-HT) pathways, including effects on key enzymes and their cofactors. Reducing the kynurenine/tryptophan ratio suggests that probiotics may contribute to balancing metabolites such as KYNA and QUIN, which may underline their neuroprotective effects and the observed improvements in cognitive function. (**b**) A potential reduction in cortical arousal states via the modulation of the brain wave percentage and power. It is suggested that probiotics may influence the transition from theta to delta waves, promoting improved sleep quality. (**c**) Modulation of neuroinflammatory pathways results from beneficial alterations to the gut microbiota. This includes enhancing intestinal barrier function, preventing bacterial translocation, attenuating the immune inflammatory response, and increasing the production of SCFAs. These SCFAs can cross the blood–brain barrier, modulating and suppressing interleukin-6 (IL-6), a cytokine associated with psychotropic effects, through the action of *Lactobacillus* species.

The strengths of this review include that it represents one of the first efforts to examine the efficacy of the genus *Lactobacillus* spp. in mood disorders. Research in this field has predominantly focused on gastrointestinal outcomes [52]. Moreover, we not only focused on anxiety and depressive symptomatology but also evaluated biomarkers associated with the GM composition, proposing potential physiological pathways underlying these effects (Figure 4).

The limitations of the present work are those inherent to a scoping review versus a systematic review or meta-analysis. Moreover, our findings demonstrate that the literature published over the past ten years still exhibits pronounced heterogeneity, precluding valid comparisons across results, particularly because mood symptomatology was evaluated using eight different instruments. This issue appears persistent in this field, as evidenced by the 2016 meta-analysis, which reported substantial heterogeneity [9]. Four different instruments were employed in the five studies included in that review, reflecting a challenge inherent to psychometric evaluation [53,54]. Similarly, it is important to note that a substantial body of evidence has been consolidated over the past decade, strengthening the positioning of research in this area across various research groups worldwide. Certain aspects, such as the dosage, intervention duration, and sample definition, have been addressed more rigorously than the syntheses published in 2016. This is reflected in the relatively low risk of bias observed in most of the publications included in the present work. It is therefore hoped that, in the coming decade, a clearer stance will emerge regarding the use of *Lactobacillus* spp.

## 5. Conclusions

The findings of this scoping review suggest that interventions employing strains of the genus *Lactobacillus* spp. hold promising therapeutic potential in modulating mood symptoms, particularly when specific strains such as *L. gasseri* or *L. plantarum* are administered in high-concentration formulations for at least eight weeks. However, the outcomes remain heterogeneous, and efficacy appears contingent upon the clinical context, the strain utilized, and whether the intervention comprises a single species or a multi-species formulation. To advance toward evidence-based recommendations regarding using *Lactobacillus* spp. as mood modulators, there is a need for the greater standardization of intervention protocols, expanded geographical representation, and long-term studies in well-defined clinical populations.

## Figures and Tables

**Table 1:** Summary of *Lactobacillus* spp. evaluated in healthy people.

Author (Year)	Country	Subjects	Probiotics	Intervention	Treatment	Mental Outcomes	Gut Microbiota Composition	Biomarkers or Metabolites	Other Results
Kelly (2017) [17]	Ireland	Healthy male volunteers (n = 29)	*Lactobacillus rhamnosus*	Capsule (1 × 10^9^ CFU) or placebo per day for 4 weeks	Unique	No significant difference between the groups	No significant difference between the groups	No significant differences were found	No observed
Nishida (2019) [18]	Japan	Healthy young adults (n = 60)	*Lactobacillus gasseri*	1 × 10^10^ bacterial cells per 2 tablets or placebo for 24 weeks	Unique	↓ STAI (*p* = 0.014) ↓ PSQI (*p* = 0.041)	CP2305 attenuates the decrease of *Bifidobacterium* spp. and increases *Streptococcus* spp.	No significant differences were found	↓ Stressful irritability (*p* < 0.001) ↓ Abdominal discomfort (*p* < 0.001) ↓ Salival CgA (*p* = 0.039)
Márquez-Morales (2021) [19]	México	University students (n = 45)	*Lactobacillus plantarum*, *Lactobacillus paracasei Lactobacillus* *brevis*	100 mL beverage (3 × 10^8^ CFU/mL) or placebo per day for 8 weeks	Unique	↓ SISCO (*p* = 0.001)	Increases Bacteroidetes and Firmicutes	Not evaluated	↓ Environmental demands (*p* < 0.001) ↓ Physical factors (*p* < 0.001) ↓ Psychological factors (*p* < 0.001)

CgA: Chromogranin A, CFU: Colony forming units, PSQI: Pittsburgh Sleep Quality Index, SISCO: Systemic Cognitivist Inventory, STAI: State-Trait Anxiety Inventory, ↓: Decreased parameter.

**Table 2:** Summary of *Lactobacillus* spp. evaluated in subjects with mood and stress-related disorders.

Author (Year)	Country	Subjects	Probiotics	Intervention	Treatment	Mental Outcomes	Gut Microbiota Composition	Biomarkers or Metabolites	Other Results
Slykerman (2017) [20]	New Zealand	Postpartum womens (n = 380)	*Lactobacillus rhamnosus*	6 × 10^10^ or placebo per day from pregnancy to 6 months postpartum	Unique	↓ EPDS (*p* = 0.037) ↓ STAI (*p* = 0.014) ↓ Rate anxiety (*p* = 0.002)	Not evaluated	Not evaluated	No observed
Lew (2019) [21]	Malaysia	Stressed Adults (n = 103)	*Lactobacillus plantarum*	2 × 10^10^ CFU/sachet or placebo per day for 12 weeks	Unique	↓ DASS42-A (*p* = 0.032) ↓ DASS42-S (*p* = 0.007) ↓ DASS42 (*p* = 0.048)	Not evaluated	↓ IFN-γ (*p* < 0.001) ↓ TNF-α (*p* < 0.001)	IFN-γ and TNF-α correlated significantly with DASS-42 scores
Ho (2021) [22]	Taiwan	Patients with chronic primary insomnia (n = 40)	*Lactobacillus plantarum*	Capsule (3 × 10^10^ UFC) or placebo per day for 4 weeks	Unique	↓ BDI-II (*p* < 0.05)	Not evaluated	Not evaluated	↓ Fatigue levels ↓ Brainwave activity ↓ Awakenings during the deep sleep stage
Zhang (2021) [24]	China	Adults with constipation (n = 82)	*Lactobacillus paracasei* strain Shirota (LcS)	100 mL of an LcS beverage (1 × 10^8^ CFU) or placebo every day for 9 weeks	Coadyuvant at depression treatment	No significant difference between the groups	LcS increased Adlercreutzia, Megasphaera and Veillonella levels and decreased Rikenellaceae, Sutterella and Oscillibacter.	Not evaluated	↓ Interleukin-6 (*p* < 0.05)
Zhu (2023) [23]	China	Anxious (n = 60) and healthy students (n = 30)	*Lactobacillus plantarum*	Powder sachet per day (1 g with 1.5 × 10^10^ CFU) or placebo for 3 weeks	Unique	↓ HAMA (*p* = 0.000) ↓ HDRS (*p* = 0.000) ↓ AIS (*p* = 0.000)	JYLP-326 restore the disturbed Bacteroides, Bifidobacterium, Prevotella and Roseburia levels	No significant differences were found	No observed
Sarkawi (2024) [25]	Malasya	IBS patients with and without subthreshold depression (n = 110)	*Lactobacillus acidophilus Lactobacillus paracasei*	2 bottles (125 mL) of cultured milk drinks (1 × 10^9^ CFU) or placebo per day of 12 weeks	Unique	No significant difference between the groups	Not evaluated	↑ Serotonine levels (*p* < 0.05)	No observed

AIS: Athens Insomnia Scale, BDI-II: Beck Depression Inventory-II, CFU: Colony forming units, DASS42: Depression, Anxiety and Stress Scale, DASS42-A: Depression, Anxiety and Stress Scale—Anxiety Score, DASS42-S: Depression, Anxiety and Stress Scale—Stress Score, EPDS: Edinburgh Postnatal Depression Scale, HAMA: Hamilton Anxiety Rating Scale, HDRS: Hamilton Depression Rating Scale, IBS: Irritable bowel syndrome, STAI: State-Trait Anxiety Inventory. ↓: Decreased parameter, ↑: Increased parameter.

**Table 3:** Summary of *Lactobacillus* spp. evaluated in MDD patients.

Author (Year)	Country	Subjects	Probiotics	Intervention	Treatment	Mental Outcomes	Gut Microbiota Composition	Biomarkers or Metabolites	Other Results
Rudzki (2019) [26]	Poland	MDD patients (n = 79)	*Lactobacillus plantarum*	2 capsules (10 × 10^9^ CFU) or placebo per day for 8 weeks	Coadyuvant at depression treatment	No significant difference between the groups	Not evaluated	↓ Kynurenine (*p* = 0.005) ↓ Anthranilic acid (*p* = 0.028)	↑ Attention and Perceptivity Test (*p* = 0.006) ↑ Californian Verbal Learning Test (*p* = 0.023)
Chen (2021) [27]	Taiwan	MDD patients (n = 40)	*Lactobacillus plantarum*	2 Capsules (3 × 10^10^ CFU) per day for 8 weeks	Coadyuvant at depression treatment	↓ HAM-D (*p* = 0.01) ↓ DSSS (*p* < 0.001)	Akkermansia, Bifidobacterium, Enterococcus, Eggerthella, Megasphaera and Ruminococcus changed significantly	Not evaluated	Coprococcus and Lactobacillus, significantly correlated with both biomarkers and depressive symptoms.
Lin (2024) [28]	Taiwan	MDD patients (n = 32)	*Lactobacillus plantarum*	2 capsules (3 × 10^10^ CFU) or placebo per day for 8 weeks	Coadyuvant at depression treatment	No significant difference between the groups	No significant difference between the groups	No significant differences were found	No observed

CFU: Colony forming units, DSSS: Depression Subscale of the Stress Scale, HAM-D: Hamilton Depression Rating Scale, MDD: Major Depressive Disorder, ↓: Decreased parameter, ↑: Increased parameter.

**Table 4:** Summary of multi-species probiotics evaluated in healthy people.

Author (Year)	Country	Subjects	Probiotics	Intervention	Treatment	Mental Outcomes	Gut Microbiota Composition	Biomarkers or Metabolites	Other Results
Rode (2022) [29]	Sweeden	Healthy adults (n = 22)	*Bifidobacterium* *longum Lactobacillus* *helveticus Lactiplantibacillus* *plantarum*	Probiotic mixture (3×10^9^ CFU) or placebo per day for 4 weeks	Unique	No significant difference between the groups	Not evaluated	Not evaluated	~ Gray matter (*p* < 0.0001)
Morales-Torres (2023) [30]	Chile	Healthy adults (n = 135)	*Lactobacillus* *helveticus Bifidobacterium* *longum*	Capsule of Cerebiome^®^ (3 × 10^9^ CFU) or placebo per day for 4 weeks	Unique	No significant difference between the groups	Not evaluated	Not evaluated	No observed

CFU: Colony forming units, ~ Stable parameter.

**Table 5:** Summary of multi-species probiotics evaluated in subjects with mood-related conditions.

Author (Year)	Country	Subjects	Probiotics	Intervention	Treatment	Mental Outcomes	Gut Microbiota Composition	Biomarkers or Metabolites	Other Results
Kazemi (2019) [31]	Iran	Mild to moderate major depressed patients (n = 81)	*Lactobacillus helveticus Bifidobacterium longum*	Sachet of Probiotic (10 × 10^9^ CFU) or Prebiotic (GOS) or placebo for 8 weeks	Coadyuvant at depression treatment	↓ BDI (*p* = 0.042)	Not evaluated	↓ Kynurenine/tryptophan (*p* = 0.048) ↑ Tryptophan/isoleucine (*p* = 0.023)	No observed
Lee (2021) [32]	Korea	Healthy adults with subclinical mental symptoms (n = 174)	*Lactobacillus reuteri* NK33 *Bifidobacterium adolescentis* NK98	500 mg capsule per day of 2.0 × 10^9^ CFU of NK33 and 0.5 × 10^9^ CFU of NK98, or placebo for 8 weeks	Coadyuvant at sleep treatment	↓ BDI-II (*p* = 0.036) ↓ BAI (*p* = 0.014)	NVP-1704 increased Bifidobacteriaceae and Lactobacillaceae, whereas it decreased Enterobacteriaceae	Not evaluated	↑ Quality Sleep (*p* = 0.006) ↓ Interleukin-6 (*p* = 0.041)
Schaub (2022) [33]	Switzerland	Patients with current depressive episodes (n = 90)	*Streptococcus thermophilus*, *Bifidobacterium breve*, *Bifidobacterium lactis*, *Lactobacillus acidophilus*, *Lactobacillus plantarum*, *Lactobacillus paracasei*, *Lactobacillus helveticus*	Vivomixx^®^ (9 × 10^9^ CFU) or placebo for 4 weeks	Coadyuvant at depressive treatment	↓ HAM-D (*p*< 0.01)	PRO increased the abundance of the genus Lactobacillus	Not evaluated	~ Inversed Simpson ~ Pielou’s evenness ~ Shannon index

BAI: Beck Anxiety Inventory, BDI: Beck Depression Inventory, BDI-II: Beck Depression Inventory-II, CFU: Colony forming units, GOS: Galacto-Oligosaccharides, HAM-D: Hamilton Depression Rating Scale. ↓: Decreased parameter, ↑: Increased parameter, ~ Stable parameter.
